# Graft materials provide greater static strength to medial opening wedge high tibial osteotomy than when no graft is included

**DOI:** 10.1186/s40634-019-0184-6

**Published:** 2019-03-28

**Authors:** James Belsey, Arnaud Diffo Kaze, Simon Jobson, James Faulkner, Stefan Maas, Raghbir Khakha, Adrian J. Wilson, Dietrich Pape

**Affiliations:** 1 Department of Sport, Exercise & Health, University of Winchester, Sparkford Road, Winchester, Hampshire SO22 4NR England; 2 Faculty of Science, Technology and Communication, University of Luxembourg, 6, rue R. Coudenhove-Kalergi, L-1359 Luxembourg, Luxembourg; 3 Department of Orthopaedic Surgery, Centre Hospitalier de Luxembourg, L-1460 Luxembourg, Luxembourg; 4 The Hampshire Clinic, Basing Road, Old Basing, Basingstoke, Hampshire RG24 7AL England; 5 Basingstoke and North Hampshire Hospital, Aldermaston Road, Basingstoke, Hampshire RG24 9NA England

**Keywords:** Tibial osteotomy, Allograft, Synthetic graft, Biomechanical analysis, Static strength, Activmotion plate

## Abstract

**Background:**

The purpose of this study was to compare the stability of medial opening-wedge high tibial osteotomy (MOWHTO) with and without different graft materials. Good clinical and radiological outcomes have been demonstrated when either using or not using graft materials during MOWHTO. Variations in the biomechanical properties of different graft types, regarding the stability they provide a MOWHTO, have not been previously investigated.

**Methods:**

A 10 mm biplanar MOWHTO was performed on 15 artificial sawbone tibiae, which were fixed using the Activmotion 2 plate. Five bones had OSferion60 wedges (synthetic group), five had allograft bone wedges (allograft group), and five had no wedges (control group) inserted into the osteotomy gap. Static compression was applied axially to each specimen until failure of the osteotomy. Ultimate load, horizontal and vertical displacements were measured and used to calculate construct stiffness and valgus malrotation of the tibial head.

**Results:**

The synthetic group failed at 6.3 kN, followed by the allograft group (6 kN), and the control group (4.5 kN). The most valgus malrotation of the tibial head was observed in the allograft group (2.6°). The synthetic group showed the highest stiffness at the medial side of the tibial head (9.54 kN·mm^− 1^), but the lowest stiffness at the lateral side (1.59 kN·mm^-1)^. The allograft group showed high stiffness on the medial side of the tibial head (7.54 kN·mm^− 1^) as well as the highest stiffness on the lateral side (2.18 kN·mm^− 1^).

**Conclusions:**

The use of graft materials in MOWHTO results in superior material properties compared to the use of no graft. The static strength of MOWHTO is highest when synthetic grafts are inserted into the osteotomy gap. Allograft wedges provide higher mechanical strength to a MOWHTO than when no graft used. In comparison to the synthetic grafts, allograft wedges result in the stiffness of the osteotomy being more similar at the medial and lateral cortices.

## Background

Medial opening wedge high tibial osteotomy (MOWHTO) is a surgical procedure commonly used for the treatment of medial varus osteoarthritis, with promising outcomes in the short- and mid-term (Laprade et al.*,* 2012; Bode et al.*,* 2015; W-Dahl et al., 2017). Performing a bi-planar osteotomy has been shown to promote bone healing with increased stability, both rotationally and antero-posteriorly (Lobenhoffer and Agneskirchner, 2003; Pape et al.*,* 2010), compared to a uniplanar technique.

A variety of plates exist for internal fixation of an osteotomy, of which the TomoFix (Synthes GmbH, Oberdorf, Switzerland) plate is considered to be the gold standard (Diffo Kaze et al.*,* 2015). Alternative internal fixators are gathering popularity with varying degrees of success with regards to clinical outcomes (pain and knee function) (Cotic et al.*,* 2015) and material properties (construct strength and stiffness) having been reported (Diffo Kaze et al.*,* 2015; Luo et al.*,* 2015). The size 2 ActivMotion HTO plate (NewClip Technics, Haute-Goulaine, France), for example, has been shown to allow a MOWHTO to resist the greatest load until failure under static compression testing, and provides the highest construct stiffness under cyclical testing, compared to the standard Tomofix plate and four other MOWHTO plates on the market (Diffo Kaze et al.*,* 2015).

Good clinical and radiological outcomes have been demonstrated when either using (Lee et al.*,* 2010; Ganji et al.*,* 2013; Saito et al.*,* 2014) or not using (El-Assal et al.*,* 2010; Floerkemeier et al.*,* 2013; Saier et al.*,* 2017) void fillers within the osteotomy gap. The different types of void filler that are most commonly used during MOWHTO can be divided into three general categories: autograft, allograft, and synthetic bone fillers. Previous research has shown that allograft and synthetic bone wedges have a similar outcome with regard to delayed- and non-union (Lash et al.*,* 2015; Slevin et al.*,* 2016) but differ in relation to other complications, for example, the use of an allograft wedge runs the risk of disease transmission, whereas synthetic grafts have been associated with infection and soft-tissue irritation (Han et al.*,* 2015).

To date, there has been one study (Takeuchi et al.*,* 2010) comparing the biomechanical properties of synthetic grafts versus no graft in MOWHTO, which found that synthetic grafts provide more axial stability to a tibia following MOWHTO. However, to our knowledge, the variations in the biomechanical properties of different graft types, regarding the stability they provide a MOWHTO, have not been previously investigated. Understanding these differences will help to inform clinical practice with regard to selecting an appropriate graft type depending on certain patient demographics. For example, patients who exert high forces through their knee, due to factors such as high body mass or physical activity levels, may benefit from a MOWHTO that confers greater resistance to mechanical loads. This could potentially reduce the risk of post-operative complications such as loss of correction, which is associated with an inferior clinical outcome (Spahn et al., 2006).

The purpose of this study was to investigate the static strength (load to failure, stiffness and valgus malrotation) of MOWHTO with inserted allografts, synthetic grafts, or no grafts. It was hypothesised that: 1) both graft types would provide greater static strength to a MOWHTO compared to when no graft was used, and 2) there would be a difference between the two graft types.

## Methods

Fifteen medium-size 4th generation analogue composite tibiae (Sawbones, Pacific Research Laboratories, Inc., Vashon Island, Washington, USA) were used. These sawbone models have been validated and shown to have similar biomechanical properties to natural human bone while having significantly lower inter-specimen variability in comparison to cadaveric specimens (Heiner, 2008; Gardner et al.*,* 2010).

### Specimen preparation

**Fig. 1:**
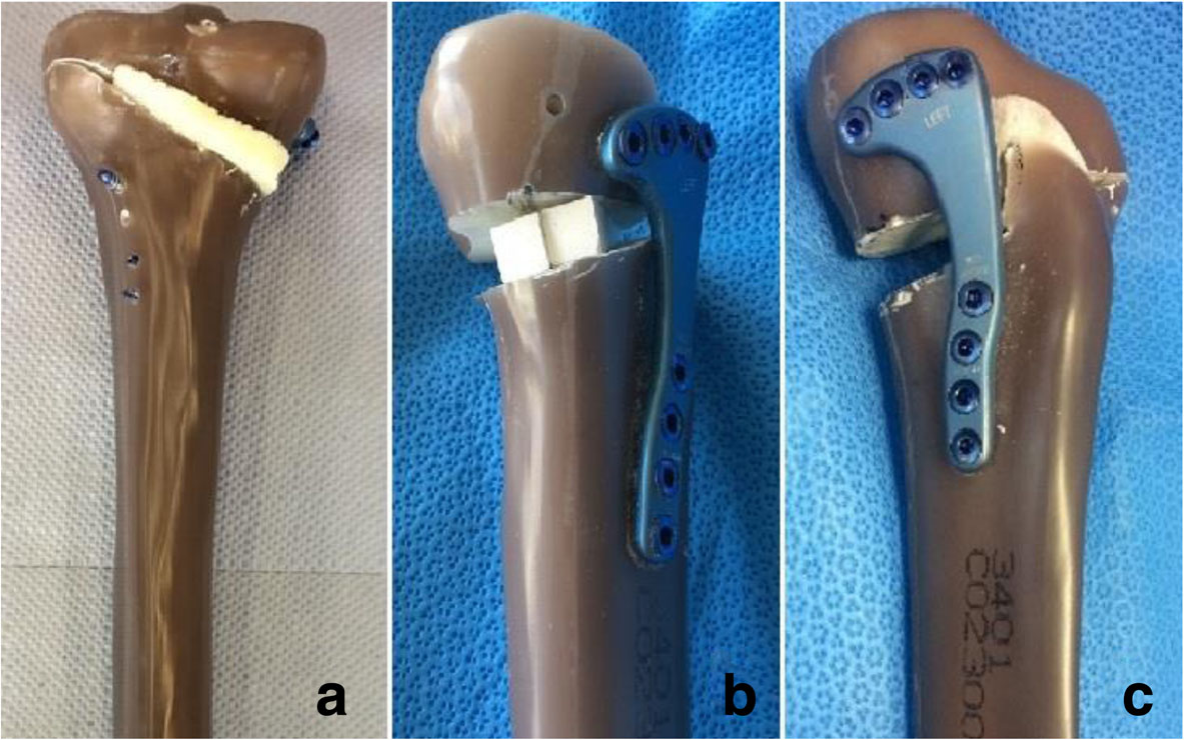
Example specimens from each test group. **a** Allograft Group, **b** Synthetic Group, **c** Control Group

A 10 mm MOWHTO was performed on each specimen by an experienced orthopaedic surgeon and fixed with a size 2 ActivMotion HTO plate (NewClip Technics, Haute-Goulaine, France) positioned antero-medially on the tibial head. Each osteotomy was performed in the same way, using the biplanar technique, with the plate being fixed according to the standard technique of the implant, as has been done in previous research (Diffo Kaze et al.*,* 2017). In five of the tibiae, a 10 mm (height) x 72 mm (depth) bone wedge allograft (RTI Surgical Inc., Alachua, Florida, USA), sourced from the proximal tibia of a donor, was inserted prior to the fixation of the plate (Allograft Group) (Fig. 1a). The width of each allograft wedge was cut in order for the graft to match the size of the osteotomy gap, as would be the case in-vivo. In the synthetic group, two 10 mm × 10 mm × 50 mm β-tricalcium phosphate wedges (OSferion60, Olympus Terumo Biomaterials, Tokyo, Japan) were inserted into another five sawbone tibiae, prior to plate fixation of the osteotomy (Fig. 1b) as has been done in previous research (Takeuchi et al.*,* 2010). Each allograft and synthetic bone wedge was held in place using an ethyl cyanoacrylate glue to prevent the risk of them slipping or falling out of the osteotomy gap during testing. The remaining five tibiae had no graft inserted into the osteotomy gap (Control Group) (Fig. 1c).

Each specimen was then prepared for testing using a previously described method (Maas et al.*,* 2013; Diffo Kaze et al.*,* 2015). Each specimen was cut 300 mm distal to the tibial plateau and placed inside a deep cylindrical mould. A scaffold was then mounted around the mould, with a centrical pinion on the inside base of the mould to ensure that the specimens were identically positioned in each case. A two-part polyurethane casting resin (FC-52, Huntsman Advanced Materials GmbH, Basel, Switzerland), created by mixing equal parts of an isocyanate and a polyol, was then poured into the cylindrical mould to better secure the specimen. Once the resin had hardened, the scaffold was removed and the specimens were rotated 180°, allowing the tibial head to be placed inside a shallow cylindrical mould, in which more of the casting resin was then poured. Before the resin was added to the shallow mould, two small metal plates were appended to the medial and lateral sides of the mould, to which the displacement sensors were then attached during testing. Finally, a custom-made sensor clamp was attached to the tibial shaft for the vertical displacement sensors to be held in place.

### Test protocol

**Fig. 2:**
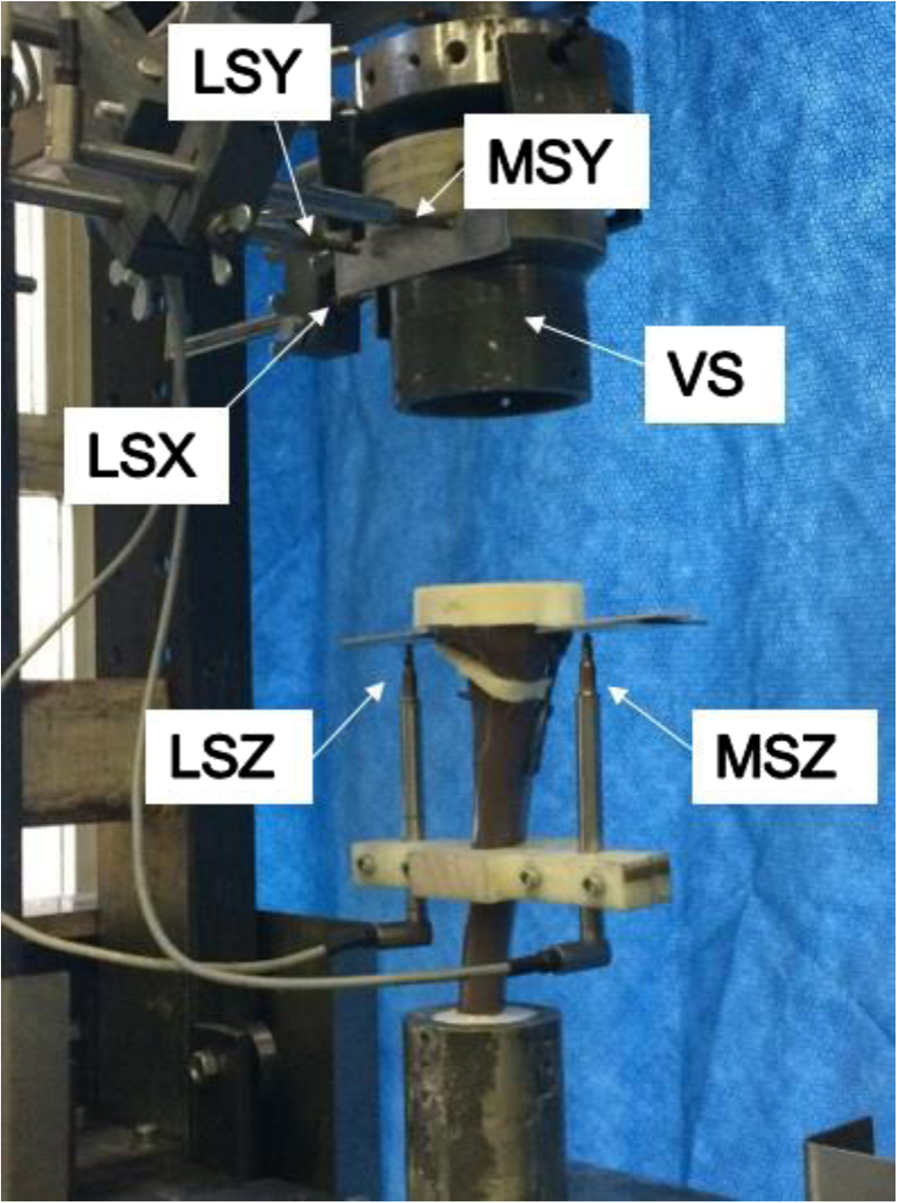
Positioning of displacement sensors around the tibial head (posteromedial view). VS = Vertical Sensor; LSX = Lateral Sensor X-Axis; LSY = Lateral Sensor Y-Axis; MSY = Medial Sensor Y-Axis; LSZ = Lateral Sensor Z-Axis; MSZ = Medial Sensor Z-Axis

Using a previously published protocol (Maas et al.*,* 2013; Diffo Kaze et al.*,* 2015), each specimen was loaded onto a 10kN hydraulic piston (INSTRON, Darmstadt, Germany), which applied a pure vertical load to the tibial head through a freely moveable support. The support was limited to only move freely in the transverse plane by the use of three freely moving metal balls. The distal end of each specimen was affixed to the piston, preventing any movement of the deep cylindrical mould in the transverse plane. Six displacement sensors were used in order to capture the deformation of each specimen at different positions around the tibial head during each test. With reference to the transverse plane, five of the displacement sensors were positioned as follows: lateral to the tibial head in the x-axis (LSX); medially and laterally to the tibial head in the y-axis (labelled “MSY” and “LSY” respectively); and medially and laterally to the tibial head in the z-axis (labelled “MSZ and LSZ” respectively). A final, vertical displacement, sensor (VS) was contained within the testing machine and measured the vertical displacement of the hydraulic piston (Fig. 2).

**Fig. 3:**
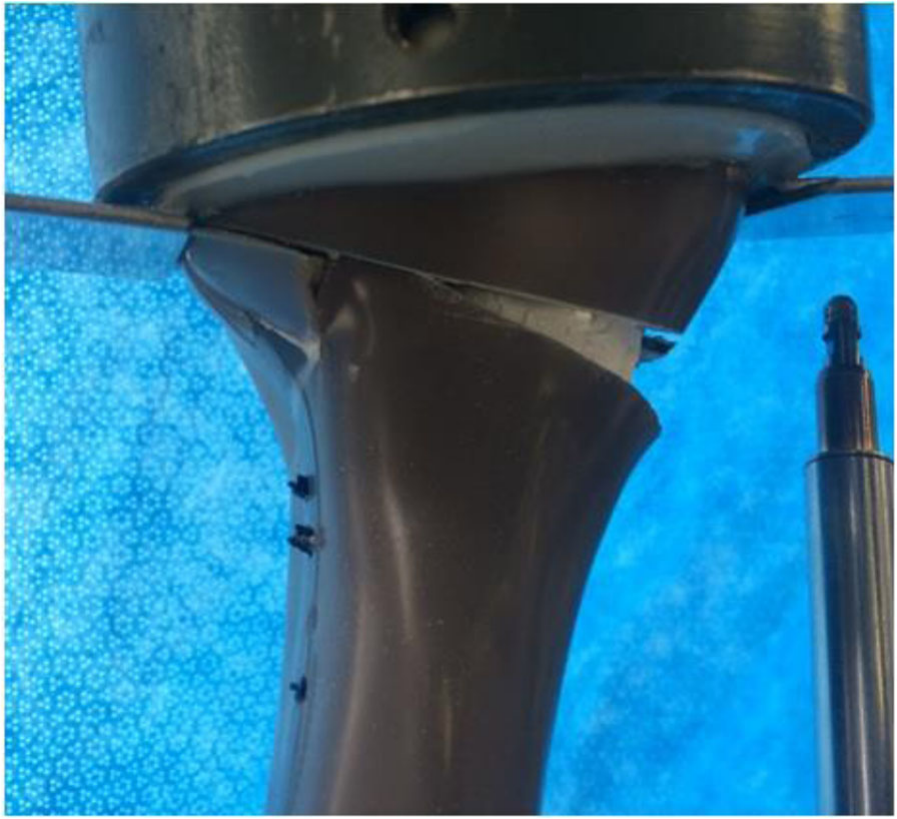
Example of lateral cortex fracture and osteotomy collapse

Once the setup was complete, the piston applied static compression to the specimens under displacement-controlled conditions with single loading to failure at a speed of 0.1 mm·s^− 1^. Failure was determined as being the point at which a simultaneous audible and visible collapse of the lateral cortex of the tibial head occurred (Fig. 3), which has been described in previous research as a “Type 2 Failure” (Diffo Kaze et al.*,* 2015). In all cases, the point of failure was also signalled by a sudden drop in the force being applied to the tibial head by the piston as the collapse occurred.

### Analysis

Due to the small sample size, statistical tests were not performed on the data and only the means are presented. However, it should be noted that the group sizes were similar to, or greater than, those in the related literature (Takeuchi et al.*,* 2010; Diffo Kaze et al.*,* 2015). The peak force (kN) and displacement (mm) of all sensors at specimen failure were recorded. Displacements were recorded as either positive or negative values, indicating direction of the displacement. Following the protocol of a previous study (Diffo Kaze et al.*,* 2015), the stiffness (kN·mm^− 1^) of each specimen (at each of the sensor positions) was calculated using the ratio of the measured force and displacement at the point of failure. Prior to calculating stiffness, any negative displacement values were multiplied by − 1 in order to make them positive so that only the absolute values were used, since the direction of each displacement is irrelevant for this calculation. Additionally, valgus malrotation of the tibial head in the frontal plane was calculated using the following formula from a previous study by our group (Diffo Kaze et al.*,* 2015):

$$ \alpha =\frac{\left|{d}_L-{d}_M\right|}{D} $$

Where “α” is the valgus malrotation (rad), “dL” is LSZ displacement (mm), “dM” is MSZ displacement (mm), and “D” is the distance between the two sensor positions. The value α was then converted from radians to degrees by multiplying α by 180°/3.14 rad.

## Results

The data from one specimen in the allograft group and one specimen in the synthetic group were not included in the final analysis due to the specimens accidentally being loaded prior to testing, resulting in the specimens failing abnormally early during their tests. Four specimens in the allograft group, four in the synthetic group, and five in the control group were analysed. All specimens, except for two in the allograft group, experienced an intra-operative lateral hinge fracture.

During testing, despite intra-operative hinge fractures occurring in most cases, all specimens failed in similar fashion due to a fracture of the lateral cortex of the tibial head (Fig. 3). Prior to failure, cracks were observed in the bones for all specimens, apart from two in the control group. Table 1 shows the mean force (kN) and time (s) at the point of failure for each group. The synthetic group failed at a higher ultimate load than the allograft and control groups, respectively.

**Table 1:** Mean force/standard deviation (kN) and time (s) at point of failure

Group	Mean Force (kN) at Time of Failure (±SD)	Time (s) until Point of Failure
Control	4.5 (±1.6)	20
Allograft	6.0 (±1.8)	25.8
Synthetic	6.3 (±2.4)	37.9

**Fig. 4:**
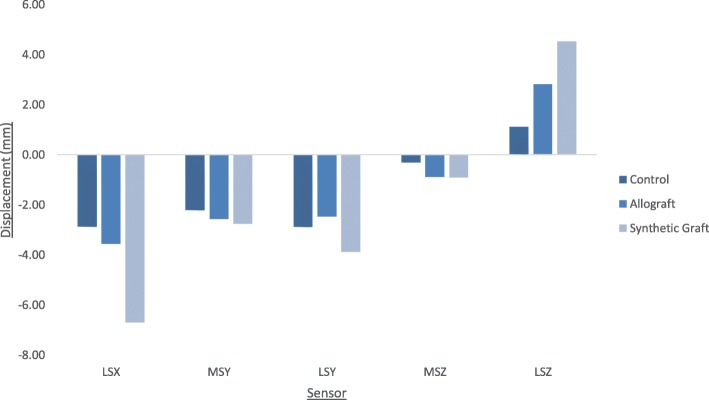
Mean displacement (mm) at each sensor position around the tibial head. Negative LSX values indicate latero-medial movement; negative MSY and LSY values indicate a posteroanterior movement; negative MSZ values indicate upward vertical movement; positive LSZ values indicate downward vertical movement. Allograft Group (*n* = 4), Synthetic Group (*n* = 4), Control Group (*n* = 5)

**Fig. 5:**
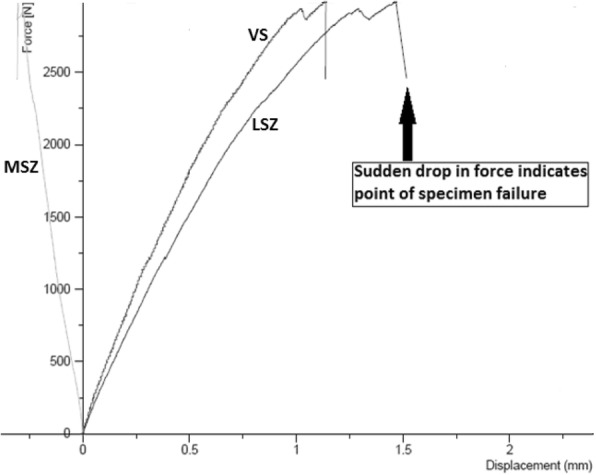
Graph showing example of vertical displacements. “VS” is positive, indicating the downward vertical motion of the piston. “MSZ” is negative and “LSZ” is positive, indicating a valgus malrotation of the tibial head

Figure 4 shows the mean displacements at each sensor position around the tibial head. A lateral-medial displacement of the tibial head during testing, as shown by a negative LSX value, was observed across all groups. Mean displacement values at positions LSY and MSY (anterior proximal tibial head) were negative, indicating an overall posteroanterior movement of the tibial head. The smallest difference between values at LSY and MSY was seen in the allograft group, followed by the control group and the synthetic group respectively. Additionally, in each group the mean absolute vertical displacement at the lateral cortex of the tibial head, position LSZ (1.1 mm, 2.8 mm, and 4.5 mm in the control, allograft, and synthetic groups respectively), was greater than at the medial cortex, position MSZ (− 0.3 mm, − 0.9 mm, and − 0.9 mm in the control, allograft, and synthetic groups respectively). When considering a downward vertical displacement as positive, values recorded at the lateral cortex were negative and values recorded at the medial cortex were positive (Fig. 5), indicating a valgus malrotation of the tibial head across all groups. This valgus malrotation of the tibial head was also measured, with the allograft group exhibiting the highest value (2.6°), followed by the synthetic group (1.8°) and the control group (0.7°).

**Fig. 6:**
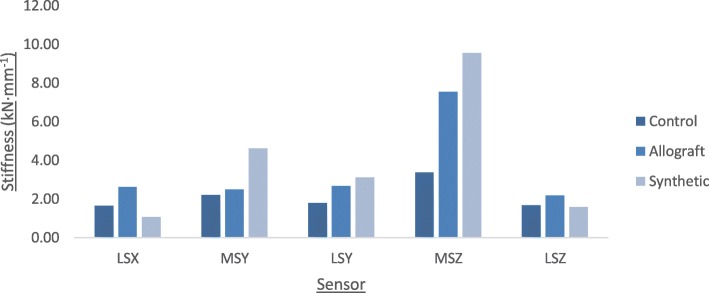
Graph showing mean stiffness at each sensor position for all groups at time of failure. Allograft Group (*n* = 4), Synthetic Group (*n* = 4), Control Group (*n* = 5)

Figure 6 shows the mean stiffness for each group at each sensor position around the tibial head. The lateral side of the tibial head was stiffest in the allograft group and weakest overall in the synthetic group, except for position LSY, where the synthetic group showed higher stiffness. The synthetic group was also the stiffest at the medial positions around the tibial head, followed by the allograft group and control group, respectively.

## Discussion

The key finding of this study is that a MOWHTO with a void filler is able to withstand more vertical compressive force than a MOWHTO without any filler, suggesting that more stability and mechanical strength is given to the knee when the gap is filled. This may have important implications for post-operative rehabilitation programmes that encourage early full weight-bearing. Although early full weight-bearing after surgery has shown promising results in tibial osteotomies with (Takeuchi et al.*,* 2009; Brinkman et al.*,* 2010; Hernigou et al.*,* 2015) and without (Brosset et al.*,* 2011; Schröter et al.*,* 2017) bone grafting, the added stability that a graft provides may reduce the risk of correction loss. Previous research (Coventry et al., 1993) suggests that there is a substantial risk of construct failure if the intended angle of the osteotomy is not accurately achieved. It can therefore be inferred that a loss of correction, or deviation from an accurate correction, should be avoided. This would appear to possible through the use of graft materials when considering the results of the present study. Additionally, a further study (Spahn et al., 2006) found that patients who suffered a loss of correction after surgery had an inferior clinical outcome, according to the Knee Osteoarthritis Outcome Score, compared to patients who exhibited no post-operative change in correction angle.

To date, only one study has compared the biomechanical effects of synthetic augmentation in MOWHTO against controls with no graft (Takeuchi et al.*,* 2010). While a direct comparison of results between that study and the present one is not possible, due to the many differences between the methods employed and materials used, the findings are similar. Both studies demonstrate that synthetic grafts provide higher stability to a MOWHTO under static compression than when no graft is inserted into the osteotomy gap. There was, however, no comparison made with the use of the allograft in that study, which has been done in the present article.

Previous studies have shown that in level walking (Morrison, 1970), an axial force of around three times bodyweight is applied through the knee (Taylor et al.*,* 2004; Heinlein et al.*,* 2009). Although osteotomy failure is not certain when no graft is inserted into the gap, based on the results of the present study, the use of a void filler with the Activmotion plate provides added stability, and may help to reduce the risk of failure during physical activity while healing is still taking place. This may be particularly relevant for obese patients (Meidinger et al.*,* 2011), or in patients who are likely to perform physical activities that may be more vigorous than level walking (e.g. stair climbing), because of the added forces that are exerted through the knee. Absolute forces of around 5.5 times bodyweight have been shown to be exerted through the knee during activities such as stair climbing (Taylor et al.*,* 2004). When considering that the control group in the present study failed at a mean load of 4.5 kN, in-vivo, a patient with a mass of 95 kg (0.93 kN) would exert a force through their knee that exceeds this level (4.65 kN) and therefore would be at risk of causing the osteotomy to fail.

Displacement values at all sensor positions, with the exception of sensor LSY on the lateral side of the anterior proximal tibial head, showed the overall lowest displacement in the control group and the overall highest displacement in the synthetic group. This is likely due to the differences in the mean length of time-to-failure between groups. Due to the displacement controlled nature of the test protocol (with the piston moving at a rate of 0.1 mm·s^− 1^) it is logical to expect that longer tests will result in larger displacement values across each of the sensors. This does, however, show that the tibial head rotates and moves more in multiple directions as higher forces are applied to it. This may suggest that providing as much stability as possible to an osteotomy could be important in the early stages of the healing process, particularly if a patient is fully weight-bearing.

The differences in vertical displacement values of the lateral and medial cortices, despite being compressed evenly by the piston, was due to the presence of the size 2 Activmotion plate on the medial side. The plate did not lose its shape or weaken when the force was applied to it. Conversely, the lateral cortex of the tibial head had no such support, which caused this discrepancy in displacement between the two sides, as the lateral cortex is the weakest point of a MOWHTO (Maas et al.*,* 2013; Watanabe et al.*,* 2014; Diffo Kaze et al.*,* 2015). Furthermore, the positive values at position LSZ and the negative values at position MSZ indicate a valgus malrotation of the tibial head. Previous studies conducting a similar test protocol on MOWHTO without grafting have reported the same findings (Maas et al.*,* 2013; Diffo Kaze et al.*,* 2017).

The highest valgus malrotation of the tibial head in the frontal plane prior to failure was observed in the allograft group. This is also the only group in which not all specimens experienced an intra-operative lateral hinge fracture. According to previous research (Maas et al.*,* 2013) an intra-operative fracture of the lateral hinge occurs frequently and is inevitable in larger corrective osteotomies (> 8°); since the specimens in the present study were corrected 10°, the number of hinge fractures prior to testing is not surprising. The fact that half of the specimens in the allograft group did not experience intra-operative hinge fracture seems to be the only difference between the groups that may explain the increased level of valgus malrotation, although this does require further investigation. Since the synthetic group withstood higher peak forces and exhibited less valgus malrotation than the allograft group, it can be said that the use of synthetic grafts reduces correction loss in MOWHTO.

Regarding the mean stiffness of specimens, the highest values were recorded on the medial side in each of the groups, which is to be expected due to the extra support provided by the size 2 Activmotion fixation plate. Highest stiffness at point MSZ was seen in the synthetic group (9.54 kN·mm^− 1^), followed by the allograft group (7.54 kN·mm^− 1^) and the control group (3.37 kN·mm^− 1^). At point LSZ, stiffness values were lower than at point MSZ in all groups, with the highest value being recorded in the allograft group (2.18 kN·mm^− 1^) followed by the control group (1.67 kN·mm^− 1^) and the synthetic group (1.59 kN·mm^− 1^). This large difference within the synthetic and allograft groups between the two opposite cortices of the tibial head is likely to be due to the volume of the graft being much larger medially than laterally, in order to fit the shape of the osteotomy wedge. The synthetic group showed the highest stiffness medially but the lowest stiffness laterally, suggesting that this type of graft offers the least support to the lateral cortex, the weakest point of a MOWHTO (Maas et al.*,* 2013; Watanabe et al.*,* 2014; Diffo Kaze et al.*,* 2015). This is further supported by the observed breaking up of the synthetic grafts at the lateral side during compression. Such concerns over the performance of synthetic grafts under compressive loads have been discussed in previous research (Amendola and Bonasia, 2010).

The allograft group showed the highest lateral stiffness and high medial stiffness. This suggests that this graft type provides more rigidity to the construct than an osteotomy with no graft, and that the stiffness is more evenly distributed than when synthetic grafts are used. It is possible that this finding could be attributed to the difference in shape of the two graft types. The allograft wedges spanned the height, width, and depth in the sagittal plane of the osteotomy gap, whereas the synthetic grafts did not fully span the width of the opening (see Fig. 1). Despite these findings, the synthetic group withstood higher peak forces before failure of the osteotomy construct than the allograft group, which may suggest a link between high medial stiffness and the maximum force required to cause a fracture of the lateral cortex of the tibial head. However, there does appear to be a limit to the beneficial amount of stiffness within a MOWHTO construct, since a certain elasticity is required to promote osteogenesis (Staubli and Jacob, 2010) and too much stiffness can have deleterious effects on bony union (Röderer et al.*,* 2014). This notion is further supported by research showing that the current “gold standard” MOWHTO plate fixator is not the one, which provides the highest construct stiffness (Maas et al.*,* 2013; Diffo Kaze et al.*,* 2017).

One limitation of this study was that it was conducted in-vitro, and without the presence of soft tissue elements, however the artificial sawbone tibiae that were used have been shown to be biomechanically similar to human bone (Heiner, 2008; Gardner et al.*,* 2010). Allografts of the proximal tibia of a donor were used in the present study. Using allografts that were retrieved from the femoral head would have led to similar results since the bone of the femoral head and proximal tibia shows similar material properties. Additionally, the loading of each bone during the tests has been shown to correspond to the loading of the lower limb in-vivo at about 18% of the gait cycle with around 22 degrees of knee flexion (Diffo Kaze, 2016). Therefore, these results can be said to approximate in-vivo efficacy. An additional variable that may have influenced the results, is the difference in shape and size of the two graft types. The allograft wedges were of a size that filled as much of the osteotomy gap as possible, whereas the synthetic grafts did not span the entire width of the osteotomy gap in the sagittal plane. Also, as a result of the low sample size, statistical analyses were not relevant in the present study. This means that the transferring of these findings to a clinical setting should be done so cautiously. However, the sample size used is reflective of previous studies (Takeuchi et al.*,* 2010; Diffo Kaze et al.*,* 2015; Diffo Kaze et al.*,* 2017).

Finally, although intra-operative lateral hinge fractures were a confounding variable in the present study, it is not expected that this had a significant effect on the results regarding the load at failure or construct stiffness. This is due to the fact that the hinge was compressed together prior to, and during testing, meaning that no excess movement of lateral part of the construct could occur. Additionally, all specimens that suffered an intra-operative hinge fracture still failed during testing due to further fractures of the lateral cortex (Fig. 3). These fractures occurred in a similar manner to those in the two specimens that did not experience an intra-operative lateral hinge fracture. Bone grafting of any sort during MOWHTO is often reserved for larger osteotomies of 10 mm or more (Aryee et al.*,* 2008; Ozalay et al.*,* 2009), hence why 10 mm osteotomies were investigated in the present study. This did, however, increase the likelihood of an intra-operative hinge fracture occurring (Miller et al.*,* 2009), so the high incidence of this in the present study was not unusual.

Conclusions drawn from the present study should only be used as a general indication of graft performance.

## Conclusion

The use of graft materials as void fillers in the gap of a MOWHTO provides greater stability and strength to the construct, compared to when the gap is left unfilled. During testing, all specimens failed due to a fracture of the lateral cortex of the tibial head. Synthetic OSferion60 wedges provide the greatest strength to a MOWHTO. Allograft HTO wedges provide superior mechanical strength and stiffness compared to MOWHTO without graft materials.

## Abbreviations

LSX: Lateral Sensor X-Axis
LSY: Lateral Sensor Y-Axis
LSZ: Lateral Sensor Z-Axis
MOWHTO: Medial Opening Wedge High Tibial Osteotomy
MSY: Medial Sensor Y-Axis
MSZ: Medial Sensor Z-Axis
VS: Vertical Sensor
